# Selections and Abstracts

**Published:** 1906-08

**Authors:** 


### SELECTIONS AND ABSTRACTS.

### The Treatment of Pruritus Ani.

BY FREDERICK S. MACY, M.D.,
United States Army, Port Adams, R. I.

The mystery enveloping certain cases of pruritus ani offers a barrier to rational treatment that renders relief frequently as much a matter of accident as of skill. Naturally, the cause determines the treatment; but, in the class of cases I have mentioned, therein often lies the mystery. Later I shall speak of conditions that penetrate the obscurity in some cases, if not all.

When the condition is dependent upon gastro-intestinal disorders such as infection by parasites, the excoriating discharge of irritants such as the products of neoplasms, ulcerations, fermentations, old dysenteries with or without fluid evacuations, the remedy is, clearly, to remove the cause or the antecedent affections. I examine the stools, therefore, as a matter of routine, microscopically. Sometimes I find evidences of some such condition as I have suggested. A vermifuge for the parasites, 30 c. c. of olive oil thrice daily for the old dysentery, or some other line of treatment appropriate to the circumstances will almost always end the difficulty. To indicate a definite mode of treatment in such instances, where the individual and the manifestations of his disorder peculiar to the particular case are the governing factors, is clearly impossible. The same is true of pruritus due to some herpetic difficulty, or to abnormal vaginal or uterine discharges, which act sometimes reflexly, and at times through the direct contact of an irritant, such as an acid, excoriating leucorrhea with the entegument. No empiric formula would be applicable to any of these cases. They must be treated individually. Hence I presume that the question is asked in reference to the obscure cases I have mentioned, for which there appear to be no assignable causes.

In regard to these, it will be found that fissures are almost always present in cases of pruritus, and are a frequent, if not the usual cause even when so small as to be almost undiscoverable. There is often present, I am tempted to say always present, so frequently is it true, a tight sphincter and stubborn constipation. These conditions exist so frequently in cases to which other causes can be assigned, that I follow the plan of treatment I shall outline in nearly all instances, as an adjunct if nothing more.

Now, it is not very sensible industriously to paint the parts, like a billboard, with solutions of silver nitrate, menthol, cocaine, or some other of the usual lotions, or to apply astringent ointments, such as the metallic oxids, and expect permanent relief in the presence of a tight sphincter. On the other hand, it is equally unwise to divulse the sphincter. That mechanism has a most useful duty to perform, and one would indeed be desperate before he would substitute one evil for another, even though these cases are usually chronic. I suggest, therefore, the following plan:

Have the patient insert into the anus as large a dilator as it will comfortably accommodate, immediately after retiring. It should be retained at least fifteen minutes. Let him do this every night until the muscles have relaxed sufficiently to receive a larger size. This treatment should be continued, as in other instances of gradual dilatation, until the sphincter is considerably looser than it is intended ultimately to be. This method, unlike divulsion, does not menace the integrity of the structure. Unless there be some rectal constriction, the small, blunt, hard rubber instruments found in the shops in graduated sizes are all that is necessary, and are inexpensive. In cases where the skin about the anus is not as well nourished as it should be, or when there are nervous or infectious complications, I supplement this treatment by introducing a tampon once daily after a stool, of ichthyol ten per cent. in glycerin solution, if there is constipation, or in a lanolin menstruum if there is not, well up into the rectum. This is best done through the speculum. Though the latter may cause some pain at first, the insertion of the instrument is itself an aid to the treatment, and the patient will tell you he feels better for it.

Relief from his method has been immediate in all the cases I have so treated. The fissures heal, when there are any, and the condition of chronic constipation is much benefited. The itching ceases with the insertion of the dilator. Of course, it recurs in the intervals between the application of the instrument, but with less severity and less frequency day by day, until it disappears altogether.

I seldom have occasion to use local applications, except, of course, for definite lesions. For these some astringent dusting powder or other medication appropriate to the case may be necessary. Equal parts of the fluid extract of hamamelis and the tincture of iodine are a combination with a wide range of usefulness. Applied locally with a camel's hair brush, it stimulates the circulation in the part, is nutritive and alterative. The fluid extract of hamamelis, in particular, appears to be of more use about the perineum and the rectum than elsewhere about the body. I advise the patient, also, to bathe the parts frequently during the day with cold, even iced water, with or without the addition of an antiseptic, according to circumstances. When the pruritus is so intolerable as to require instant attention, I find that a solution in water of bichlorid of mercury, one part in five hundred, applied ice cold and instantly washed away with clear iced water, will give relief when other means fail. Menthol in alcoholic solution, 0.64 gramme in 30 c. c., is also useful. I have already mentioned several other local applications, but, as I have said, I seldom find it necessary to use them. Inserting the speculum or a suitable dilator will often give as immediate and as great relief. —*New York Medical Journal, April* 28, 1906.

---

### THE MODERN MANAGEMENT OF MALARIAL ANEMIA.

One of the most obstinate forms of anemia with which the physician has to contend is that which succeeds malarial infection.

This particular form of anemia is, unquestionably, due directly to the structural changes induced by the protozoon parasite.

While a mild form of anemia is a common, if not invariable, consequence of malarial infection, there is a severe type, termed *malarial anemia*, which not infrequently occurs. This latter variety usually responds slowly to curative measures; and, since its existence renders the individual a fit subject for recurring malarial manifestations upon the slightest exposure, the importance of its cure can not be too strongly emphasized.

The doctrine of the latency of malarial poisoning in the human body is rapidly gaining in popularity. Some authorities even go so far as to claim that a person who has once been inoculated with the malarial protozoa never completely recovers.

Whether this be true or not, it is certain that the protozoon parasite does exert an influence which tends, for a great length of time, to lower vitality and render feeble the powers of resistance to renewed attacks. This is especially true in the case of women, children and persons of advanced age.

Recent investigators unite in ascribing the cause of *malarial anemia* to the liberation of hemoglobin from the red corpuscles in the blood vessels. The pigmentation resulting from this liberation of hemoglobin is one of the characteristics of malarial infection. And while the coloring matter may remain in the blood stream, it usually infiltrates into the cells and neighboring tissues. The deposit of pigment is especially great throughout the tissue of the liver and spleen.

The thickening and softening of the mucous membrane of the stomach which always attends malarial infection, seems likely to contribute, at least to some extent, to the development of anemia.

In every instance the degree of the anemia is in direct ratio to the amount of the hemoglobin liberated from the red corpuscles. And this fact explains the philosophy of effecting repair by the administration of iron, the hemoglobin-contributor.

Whether or not the protozoon parasite is ever completely eliminated from the economy remains an unanswered question. But it is now universally conceded that the protracted administration of iron does render the individual partly, if not completely, exempt from a return of malarial manifestations of an aggravated type. Far more so, in fact, than does quinine. Indeed, we have good cause to believe that iron does exert a destructive influence upon the malarial protozoa and increases the immunity of the individual.

While it is the chief aim of the physician to make up the deficiency of the hemoglobin in these subjects by the administration of iron, it is distinctly important, coincidently, to increase the appetite and augment the capacity to appropriate the food ingested.

To this end, discrimination in the selection of the form of iron to be employed is vitally essential. The acid solutions of the drug are ineligible because of the fact that they can not be engaged for a long period without harmfully affecting the secretion of the digestive juices and adding to the morbid state of the mucous surfaces of the alimentary tract.

Furthermore, the continued use of acid products of any sort are certain to diminish the alkalinity of the blood, thus depressing, to a very considerable extent, the nutritive processes. Then, too, headache, which is an ever-disturbing factor in these cases, is intensified by all substances of an acid reaction.

The strongly alkaline preparations of iron, while less objectionable than the acid ones, are open to fault for the reason that they induce constipation, and in this manner favor autointoxication.

By far the most effectual form of iron in the treatment of *malarial anemia* is that which is neutral in reaction and available for immediate absorption. The organo-plastic form of iron, as found in Pepto-Mangan (Gude), certainly fulfils the requirements of the physician with greater promptness and uniformity than any other product thus far evolved.

This preparation—Pepto-Mangan (Gude)—is by all means the most potent hemoglobin-producing form of iron, and it undoubtedly surpasses other ferruginous products as an invigorator of the digestive and nutritive functions. These assertions are easily confirmed by the microscope.

It is also an accepted fact that Pepto-Mangan (Gude) does not induce constipation, and it seems to materially hasten repair of the mucous surfaces of the alimentary tract resulting from the structural changes incident to the malarial infection.

In short, Pepto-Mangan (Gude) is of inestimable value in the treatment of *malarial anemia* by virtue of its manifold advantages over other preparations of iron.

If this preparation is administered for the proper length of time, the individual gains substantially in strength, flesh, physical and mental energy.

---

### THE TREATMENT OF WHOOPING COUGH.

BY R. F. LEFEVRE, Reading, Pa.

The treatment of whooping cough may properly be divided into these measures, viz.: Prophylactic, hygienic and medicinal.

*Prophylaxis.*—Whooping cough being a highly contagious disease, spreads by contagium in the great majority of cases, and only rarely miasmatically, the first essential in treatment is prophylactic.

The avoidance of contact with whooping cough patients is very important. It is sometimes deemed wise by physician and parents during mild epidemics not to isolate children too carefully, especially if any one member of the family has already been attacked with the disease, so that they may be immune to a future, and maybe more severe outbreak. Still this should not apply to very young children, under four years, or children of consumptive parents, or scrofulous or rachitic children.

Children should be protected from taking colds as much as possible, since this predisposes the child during epidemics.

Preventive measures have failed so far, no adjunct having proved of value, if one is obliged to remain within the limit of an epidemic and of the contagium.

*Hygiene.*—Cleanliness and refinement are essentials in this disease. Great care should be exercised in taking proper precautions with the sputa and excretions in general. Schools, kindergartens, and play-grounds of children should be properly fumigated and disinfected in severe epidemics.

The diet should be one easily digested and assimilated. Proper clothes should be put on children, and an abdominal binder on small children, as a precaution against hernia during the paroxysmal stage. Properly regulated exercise and rest for the patient is advisable.

A temperate climate should be sought. If the weather is moderately warm and moist, there is no objection to open air treatment, especially after the catarrhal stage has subsided. Bowel action should be carefully watched and regulated if necessary.

*Medicine.*—The vast number of drugs employed and hailed as of benefit in this disease makes it obvious that none have proved as a specificum.

The disease having once developed, there are no drugs which will cure, shorten or modify it in the least. Of the innumerable drugs which have been recommended for this disease, four possess undoubted advantages over all others, viz.: quinine, belladonna, antipyrine and creosote.

I use as a prophylactic and tonic during the continuance of the disease the following prescription:

| | | |
|---|---|---|
| ℞ | Quininæ tannatis | gr. xxiv. |
| | Olei theobromatis | ʒvi. |
| M. | Ft. trochisci No. xxiv. | |

Sig.: One lozenge three to four times a day.

To modify the severity and relieve the frequency of the spasms during the paroxysmal stage I prescribe belladonna and antipyrine, either alone or in combination. The following prescription proved very efficacious, and is, in my opinion, the best. The dosage is for a child two to four years of age, and larger doses can be given with asserted success in many cases:

| | | |
|---|---|---|
| ℞ | Antipyrini | gr. xii. |
| | Tr. belladonnæ | m. xlviii. |
| | Syr. aurantii flor | f℥i. |
| | Aquæ | q. s. at f℥iii. |

M. Sig.: A teaspoonful every four hours.

When paroxysms are so severe and frequent as to disturb and prevent sleep, opium in some form, or nerve sedatives, may prove beneficial, such as hyoscyamus, cannabis indica, etc. These should be reserved, and only given when highly necessary, and with precaution.

Creosote I have administered with success by inhalation, vaporized over an alcohol lamp. It allays irritation, facilitates the expulsion of the mucus, and acts possibly as antiseptic. The possibility of absorption should not be forgotten, and the urine carefully watched.

There are numbers of other drugs used for this disease, but space does not permit of giving them all, and their enumeration would be only confusing. This outline of treatment, as stated, has given me most excellent results, of course modified as indicated in individual cases.—*N. Y. Med. Journal,* April 14, 1906.

---

### ACTION AGAINST PHYSICIAN FOR CONVEYING SCARLET FEVER TO A PUERPERAL WOMAN.

A remarkable case has just been decided in the courts which in consequence of its widespread importance to the profession, has attracted much atttention. A physician was called to a case of scarlet fever, after seeing which he went home and disinfected his hands. Two hours later he was summoned to a case of labor. On the fifth day the woman developed scarlet fever. The woman's husband alleged that the doctor conveyed the disease to her and claimed damages for negligence. For the plaintiff it was urged that a puerperal woman was specially liable to infection with scarlet fever in a specially malignant form, and that more complete precautions against conveying infection should have been taken. Counsel supported this view by reading extracts from the writings of Dr. Galabin and Dr. Boxall, but as he did not call these authorities, the judge ruled that this was not evidence. On the other hand, Drs. Herman and Hunter were called for the defense and stated that a puerperal woman is neither more nor less liable to scarlet fever than other people; that puerperal scarlet fever is rare, and that it is not more dangerous than scarlet fever apart from the puerperal state. The next point in the plaintiff's case was inspired by the writings of Dr. Boxall, who stated that the following precautions should be taken after visiting a case of scarlet fever, before going to a case of labor: "1, A disinfectant bath; the whole body should be immersed, the head above all, for the hair is a veritable network to entangle the poison; 2, a complete change of clothing; 3, active outdoor exercise." The physician, according to his evidence, after seeing the case of scarlet fever, walked home, a distance which occupied about five minutes. He found a message to go to the case of labor. He disinfected his hands and arms with izal and changed his coat. He visited another patient, walking about a quarter of a mile, and took a cab and drove a distance of two and one-half miles to the patient's house. He went in and shook hands with her. Then he retired to an ante-room, took off his coat, scrubbed his hands and arms with soap and water and repeated the process in a solution of corrosive sublimate, and finally rinsed them in a solution of izal. After putting on rubber sleeves, which he had previously disinfected, he examined the woman. Two physicians were called for the plaintiff and stated that a physician should transfer a case of labor to another if he was called to attend it within two hours of visiting an infectious case. This would be their own course. They considered that in order to disinfect himself a physician should change all his clothes and take a bath. For the defendant a large amount of medical evidence was called, including Dr. Herman (London Hospital), Dr. Hunter (London Fever Hospital), and Professor Spencer (University College Hospital). They all agreed that the precautions taken were the ordinary ones and quite sufficient, though there were more complete ones, such as those described, but that these were impracticable in general practice. None of these witnesses agreed with the opinions quoted from Dr. Boxall of the special susceptibility of the parturient woman to scarlet fever. The jury gave a verdict for the plaintiff.—*Journal A. M. A., March* 31, 1906.

### PETERSBURG DOCTORS AND REPEAL OF THEIR LOCAL SPECIAL LICENSE TAXES.

The Richmond physicians very recently by concerted action were enabled to show the council and board of aldermen good reason why the special license tax levied upon them for practicing medicine should be abolished, with the result—now that the usual "pay-day" for such onerous tax has rolled around—the relief afforded is more readily and peculiarly appreciated.

Doctors are commonly good citizens, and have no desire to avoid the duties and burdens in the way of taxes, or otherwise, of good citizenship. Considering the charitable character of a large portion of their work, they feel it is unjust and inequitable that a special tax should be charged them. We have never yet seen the doctor who had one word to argue against a *legitimate income tax,* poll tax, property tax, or any other sort of tax that the average citizen has to pay, but when it comes to saying that he must pay a special tax for the privilege of practicing medicine—a work in many ways not less noble and humane than the ministry of the gospel—and a tax which is in reality the equivalent to a *second income tax,* varying as it does with the physician's income, we very properly object to having this hardship forced upon us.

There are naturally some physicians so fortunate—in a small minority of cases by means acquired solely by the practice of medicine, but in the larger number of instances through other instrumentalities—as not to feel the strain upon their finances from the payment of these special taxes. While possibly such members of the profession may deem the worry of attempting to have such an ordinance abolished is not worth the bother to themselves, they undoubtedly owe something to those not so favorably situated. If the principle governing the law is wrong for the hard-working doctor whose clientele is slow pay or does not pay at all, and who has to scuffle to make ends meet, it is likewise wrong for the more successful, and he should lend what aid is at his command through his own efforts, or that of his influential patrons, to correct the existing evil. Why should the well-to-do doctor be ashamed that he or his brother had worked for the repeal of the special license tax?

Among other things, special taxes are levied on certain forms of business, as for instance, barrooms, pawnshops, and similar instances, for the purpose of limiting the number of such places, and to a certain extent to make them approach respectability as near as possible, and in some degree control the manner of their doing business. There can be no such reason governing the practice of medicine. Of all mankind, where can be found a class of business interests that *deals in philanthropy* more generally and more liberally than the doctor? What good reason can be assigned for this special license tax on him?

We can only view the matter as one of—we hope—thoughtless imposition. At any rate, wherever such laws are in force, there should all the doctors unite to have them removed. Work, and good hard work—never ceasing until the end sought has been attained—will win in Petersburg, or elsewhere. The injustice of *double income taxation* on doctors will appeal to most reasonable law-makers.—*Virginia Medical Semi-Monthly, April, 1906.*

---

### IS DOCTOR JOHN V. SHOEMAKER AN ADVERTISING PHYSICIAN?

Why do physicians advertise? Is it to gain reputation and practice or to gain notoriety?

What excuse can Dr. Shoemaker give for the article entitled "Shall We Kill Incurables?" which appeared in the *Saturday Evening Post* of March 10, 1906? Is there anything in this article that the laity need know?

Will one person out of ten rightly interpret his closing paragraph? "Meanwhile, if I were asked to define the wisest policy for the every-day person who wishes *to keep well and to avoid dying,* I would say: *Stay away from the doctors as long as you can;* use medicines only when they are necessary, and, above all, when attacked by any bodily affliction, give Nature every possible chance to assist in a cure." [The italics are ours.]

Do you, my professional brethren, know what he means by stating that if the every-day person wishes *to keep well* and *avoid dying,* he should *keep away from* the *doctors* as long as he can?

Why did not a man so learned in materia medica and therapeutics write such an article on the nostrum evil? Would such an article be less widely quoted? Would it be less apt to bring him into the limelight with Osler?

Why did he write after his name "President of the Faculty of the Medico-Chirurgical College of Philadelphia?" Was it to show the public that he is a high authority? Did he cite the supposed case of Addison's disease and that case of anemia to illustrate that, as a "young fellow," he was a brilliant diagnostician, but that now he is a "has been," who might need Oslerism?

Is this dissertation an "ad" for Aix les Bains? Did he intend to say that his observations confirmed the statement of the official, who said that all who came there get well? With what incurable diseases were these people afflicted?

Why did Dr. Shoemaker not address his remarks to the medical fraternity? Was he afraid the physicians would pay no attention to his statements? Did he think it safer to inform the public, so they would not be murdered in their beds?

Does Dr. Shoemaker say that physicians now know that diseases formerly called incurable are not always so? If physicians know this, who could be hired to kill the so-called incurables?

What physicians advocated killing so-called incurables? Why don't he favor us with their names?

Will all who read the Shoemaker article take seriously the Doctor's statements that we do not know any satisfactory test of death? Will the laity work over their decomposing dead relatives for hours and days trying to revive them before turning them over to the undertaker? T. MITCHELL BURNS.

[Since writing the above we find that extracts and comments on Dr. Shoemaker's article have been telegraphed all over the country.]—*Editorial Colorado Medical Journal, March, 1906.*

### COMMON DUCT CHOLELITHIASIS.

In *Journal of Surgery, Gynecology and Obstetrics,* W. Mayo Robson discusses the subject of common duct cholelithiasis. Many new and interesting points are brought out by the author. The author finds the occurrence of stone in the common duct in 40 per cent. of his cases. The symptoms of common duct stones are repeated "spasms" and jaundice. The jaundice is not as deep as we find in cancer of the pancreas or common duct, and is more intermitting. This changeable jaundice is caused by the movable stone in the duct acting on the principle of a ball valve. The author claims all cases of cholelithiasis are accompanied by more or less inflammation, and we frequently find as a result adhesions. The obstruction of the duct is seldom complete, and in consequence distension of the gall bladder is not always present, as we would suppose from backward pressure of bile. The muscular coat of the gall bladder contracts in an effort to expel the stones. This contraction continues until atrophy of the gall bladder ensues. The author claims that the differential diagnosis of common duct stone and cancer of the head of pancreas is not always an easy task. By some elaborate chemical examination of the urine brought out by Dr. Cammidge certain acicular, sharp-edged crystals arranged in rosettes are found. If these dissolve in dilute sulphuric acid in thirty to forty seconds impacted stone or pancreatitis diagnosis is made. If these crystals are thick and rounded and dissolve in sulphuric acid in one to five minutes cancer of the pancreas is the diagnosis. It is lamentable that the author did not in his valuable article describe this elaborate chemical process to enable others in using it in making a diagnosis. The author mentions twenty-eight different conditions that may threaten the life of a patient suffering with common duct stone. The operation is rendered easier if with a longitudinal incision the liver is turned upwards and outwards. In this rotation of the liver we bring into view the common duct and the duodenum and an otherwise difficult and deep operation is rendered more superficial and easier in execution. Drainage of the common duct by a rubber tube fastened by catgut suture in situ is used by the author, together with drainage of the kidney pouch. In other cases the incised duct is sutured with catgut and the gall bladder drained. Drainage is life-saving and very imperative in cases accompanied by much inflammation. The author further adds that when streptococci are found in the bile either in the gall bladder or ducts an unfavorable prognosis should be made. The author reports seventy-six choledochotomies with three deaths. The first from pyemia, the second from septicemia and the third from cholemia. The latter case as well as the second the author states if drainage was rigidly established the results might have been better.—*Medical Fortnightly.*

---

### CANCER OF THE RECTUM.

After a critical review of the various methods that have been devised for high cancer of the rectum, Mayo describes the operation that he practices. It is a modified form of the Quenu operation, the tumor being attacked by the abdomino-perineal route. With the patient in a high Trendelenburg position, a median incision is made. The limits and relations of the tumor having been noted, the lower end of the sigmoid, at about the level of the sacral promontory, is divided between two clamps. The proximal end is brought out of the wound and closed with a purse-string suture. A gridiron incision is then made on the right side, as for an appendicectomy, and the sutured end of the sigmoid is pulled through this opening and sutured in place, so that about three-fourths of an inch of the bowel is left projecting. The distal stump is now closed by inversion, and the bowel is carefully dissected away from the pelvic contents and sacrum down to about the point where the middle hemorrhoidal vessels supply the rectum. The entire area is now packed with moist hot gauze and the patient is placed in the perineal position. After closing the anus with a suture, a circular incision is made around the anal margin, and the rectum is dissected away from the prostate and urethra or the vagina up to the point where the dissection had been conducted above. An assistant passes the abdominal end through the perineum and the whole is removed. The peritoneal denudation in the pelvis is closed as well as possible, with drainage through the perineum. The sigmoid trap is pulled down over the exposed surface, and in the female the uterus and broad ligaments are adjusted with a few sutures to aid in covering. The abdominal wound is closed and the perineal is narrowed to the proper dimensions. The end of the sigmoid is opened at the end of twenty-four hours. The advantages of the operation are these: the radical removal it affords; the anus placed in a position that permits easy inspection and cleansing; the sigmoid trap obviates frequent stools and the intermuscular incision gives a fair degree of control. Mayo reports nineteen cases, of which five died in the first month. Of four cases that passed the three-year limit, two are alive and well.—*St. Paul Medical Journal, April,* 1906.

---

### SHALL THE INDEPENDENT MEDICAL MAGAZINE GO?

The *St. Louis Medical Review* offers some timely considerations on this question. It recognizes that organization is a very important and a very necessary thing; without it the extent of possible achievement is necessarily curtailed. But if its purpose is to crush out individualism, it will but substitute for the lesser evil of inadequate strength the gigantic one of a tyranny. Organization for defense and for scientific advancement is an admirable thing, but watchfulness is the part of wisdom, for man's nature has not appreciably changed since the dawn of history. The primal passions and emotions are to-day exactly what they were 2,000 years ago, and there is no reason to doubt that 2,000 years hence they will be exactly what they are to-day. Their mode of expression has altered, that is all. Robber barons, it is true, no longer go forth a-foraying with the strong arm to ravage and waste and plunder; but have we not trusts, corners, combines, which effect with the astute brain exactly the same ends. from the same motive—lust of power, inherent, primal, eternal as the pride of possession, the promptings of sex, the instinct of life itself? We aim these remarks at no one in particular; it is man, not men, that is to blame. When organization shall have stamped out all individualism, have suppressed all independent journalism, it will have rendered impossible for the minority all expression of dissent; then will it, perchance, be found that the guns mounted for the defense of the city have been seized and turned upon the city itself while men slept. We have been explicitly informed that "the day of the privately owned weekly medical journal is passing away. Its place will be taken by the *Journal of the American Medical Association* and the various State journals." The wish, no doubt, is father to the thought. But should it prove to be the case, then will dissentients find themselves without a medium of expression, or what is worse, because entailing a false position, with only a medium that is necessarily *ex officio* antagonistic. Each one of you who reads these lines may one day find himself by force of conviction in the dissentient ranks, and then without a rallying point, and with all the organization in his adversary's hands, then—let him remember the fable of the horse and the man!—*Medical Standard.*

---

### THE INFLUENCE OF SODIUM CHLORIDE UPON DROPSIES IN CHILDHOOD.

O. Grüner (*Weiner klinische Rundschau,* No. 12, 1906), after a number of experiments extending over several years, has determined that in kidney affections the excretion of sodium chloride is interfered with, and in consequence, in order to establish osmotic equivalence, there is developed pathological water retention and edema. Observations made with a diet deficient in chlorides, in a case complicated with heart disease in two acute and two chronic cases of parenchymatous nephritis, one interstitial nephritis, and two healthy infant (purslings) gave the following results: The cardial edema entirely disappeared, but returned in a few days to a less extent, when salt (150 grains a day) was added to the food. An ordinary mixed diet also had the same effect. The body weight increased coincidently with the retention of the chloride. In the cases of nephritis also sudden increase of the dosage of salt was attended by a corresponding increase in the dropsy and the bodily weight. In these the reduction of salt in the food did not always cause an equally prompt diminution in the edema. In the case of interstitial nephritis there was observed a lasting tendency to increased chloride excretion, but without corresponding loss of weight. It appears that just in these graver cases of disease of the kidney the retention of the chloride is not always accompanied by a corresponding degree of water retention; but may far exceed it (i. e., when considered in its osmotic equivalence). In the healthy infant, Grüner found that by suddenly increasing the daily intake of sodium chloride that there was a retention both of chloride and water causing increase of weight, owing to the inability of the kidneys to excrete the unaccustomed quantity of salt, the retention of which naturally led to the retention of water. From these considerations the practical clinical conclusion is drawn that in renal and cardiac dropsies, and where there is a tendency to their development, it is advisable to place the patient upon a diet as free as possible from sodium chloride.—*N. Y. Med. Journal, April* 28, 1906.

---

### THE EARLY DIAGNOSIS OF PULMONARY TUBERCULOSIS.

Pryor (*Med. Rec.,* November 25, 1905) quotes the following excellent definition of incipiency or favorability from a committee report of the National Association for the Study and Prevention of Tuberculosis: "Slight initial lesion in the form of infiltration limited to the apex or small part of one lobe. No tuberculous complications. Slight or no constitutional symptoms (particularly including gastric or intestinal disturbances or rapid loss of weight). Slight or no elevation of temperature or acceleration of pulse at any time during twenty-four hours, especially after rest. Expectoration usually small in amount or absent. Tubercle bacilli may be present or absent." (We would heartily advise every general practitioner to memorize this quotation; many a life may be saved by an intelligent application and appreciation of it.) Pryor, who has done much institutional work, declares his conviction that pulmonary tuberculosis is rarely recognized at an early and proper time for a successful treatment. And herein is stated a pitiful truth. Inquiries submitted to a number of sanatoria in the United States, to learn the percentage of incipient cases received, as above defined, reveals the fact that the average percentage of all patients under treatment which could be designated as early and favorable varies from two to thirty. Among reasons for this state of things are: That the patient does not seek medical advice early enough; especially is this the case among the poor, who, moreover, receive incompetent counsels when they go to a physician or dispensary. Another is the faulty education in medical schools and the recent tendency to give prominence to laboratory methods at the expense of the skill of the clinician. One finds, in addition to the symptoms stated, loss of appetite, chlorosis or anemia, loss of weight, cough, with or without expectoration, hemorrhage and fever; it is very rare that all of these are associated at an early time. They are apt to be present at a later stage—particularly in patients who have had rest, nourishing diet and open air treatment; oftentimes under these conditions, few or no symptoms are manifest, yet the disease may be progressive. The physical examination should be painstaking, with the clothing removed to the waist.

---

### CONTRIBUTIONS TO THE TREATMENT OF EPILEPSY.

By DR. STRUMPELL (*Dtsch. Arrh. f. Klin. Med.*, Vol. 84, 1905).

Spontaneous recoveries from epilepsy are not impossible but exceedingly infrequent. Certain important chapters of a life-history, such as the onset or cessation of menstruation, the beginning of old age, pregnancy, the puerperal condition, etc., all these may exercise a favorable as well as an unfavorable influence, although it has so far proved impossible to derive any fixed rule from these observations. The entire theory of reflex epilepsy needs a very careful revision. It goes without saying that every distinct simultaneous disease of a patient having epilepsy should be treated professionally as far as indicated by this associated condition (chronic nasal or aural disease, etc.) ; but no influence of this treatment upon the epilepsy should be promised. The author cautions especially against unnecessary gynecological operations; saying that female epileptics are not in as much danger from certain gynecologists as are female hysterical patients, but that many a gynecological crime has been perpetrated upon them also.

The author has no faith in a marked influence of vegetarian diet upon this disease, nor in the effect of a strict milk diet. He interdicts a very abundant supply of salt, without placing an exaggerated stress upon this rule. In all graver cases, he recommends total abstinence from alcohol, and if feasible, from nicotin also. Considerable physical exertions are by no means invariably harmful. In the case of one patient under the author's care, the disease was very favorably influenced by prolonged and exhausting mountain trips.

Personal and individual management is needed for the muscular work allotted to each patient; rest in bed not being suitable for all cases. The author's attitude towards bromide medication is to the effect that the symptomatic effect of the remedy is frequently overrated, whereas its injurious side-results are often underestimated. In the treatment of patients who have to be confined in institutions, the bromides are sure to retain their importance. In the milder cases, however, those generally treated by medical practitioners, Strümpell advocates that the use of bromides be restricted. It is absolutely unnecessary to prescribe bromides at the first attack, or in those cases where the attacks occur only at intervals of two or three months, or longer. If the remedy is employed after all, its effect upon the general condition should be carefully watched, weighing the advantages against the disadvantages. Here the subjective statements of intelligent patients form a legitimate basis. When the attacks are so frequent that their suppression is eminently desirable, it is certainly proper to try the bromide preparations in the first place. Even then, however, the condition should never be determined by the frequency of the attacks exclusively. The author says that all text-books teach that epilepsy in the majority of cases will finally lead to permanent disturbances of the mental capacities and the physical condition. He is firmly convinced that many (of course not all) of these disturbances are to be attributed, not to epilepsy at all, but to chronic bromide intoxication.

---

### TUBERCULOSIS IN SCHOOLS.

In a careful study of the schools and scholars of Paris, Dr. Vautier (*Revue d' Hygiene et de Medicine Infantiles*) arrives at the following conclusions:

"In the common schools of Paris, tubercular contagion appears to us to be very rare. This contagion may be produced in a family during the school age, but even that is not frequent. The number of children of school age, who have clearly pulmonary tuberculosis is very small. Children, in a great majority of cases, at least among the poor, are infected by latent tuberculosis at the time of their entrance into school."

He therefore proposes the following rules and regulations:

Add to the instruction given relative to the construction of school buildings a rule favoring facing the building so that the sun's rays may penetrate the class-rooms and the courts.

Replace wooden floors by those without joints in schools to be built, and if possible in those already built.

Exclude children who have evident tuberculosis, except that surgical tuberculosis may be so protected by airtight dressing as to be safe.

Send away members of the teaching force who have evident tuberculosis or put them in places where they will not come in contact with children.

Have school-rooms often thoroughly cleaned.

Seek to increase the strength of children by walks and games for which open lawns are necessary. Fortifications or the space about them are all that is necessary.

Insist that the schoolmasters advise cleanliness, and if need be, require it, and that they make it possible for them to know the meaning of a bath.

Insist on obtaining from the boards of health proper aeration and size for homes and disinfection of places occupied by bacillus-bearing tubercular patients, specially at the time between the departure of the infected family and the entrance of another family.

Invite the school boards to consider the matters of better food for the children, furnishing a meal to many of them and making arrangements for free medicines, specially those prescribed by the attending physician.

Send children convalescent from acute sicknesses, specially those likely to become tuberculous; measles, pertussis, etc., to special institutions in the country.

Establish seashore hospitals at convenient places, where tubercular children may stay six months or a year, and receive suitable treatment.

---

### PERNICIOUS VOMITING OF PREGNANCY.

In the March issue of Johns Hopkins' *Bulletin,* J. Whitridge Williams gives a most extensive and valuable review of the literature upon this subject. His object is to bring together the more important contributions to the literature, and, guided by his own experience, to set up certain well-defined types of the disease, in the hope of establishing more satisfactorily its etiology and laying down more definite rules for guidance in its treatment. From his studies the following conclusions are deduced:

The pernicious vomiting of pregnancy is not due to a single etiological factor, and occurs as one of three varieties, reflex, neurotic, and toxemic.

The reflex type is dependent upon the existence of abnormalities of the generative tract or ovum and may be cured by their correction or removal.

The neurotic type is dependent upon the existence of a neurosis without demonstrable lesions and is more or less allied to hysteria. It is the most frequent variety of serious vomiting and can be cured by suggestion or a modified rest cure.

The toxemic type is associated with characteristic changes in metabolism and in fatal cases, at least, with lesions in the liver analogous to those observed in acute yellow atrophy. It may occur in an acute or chronic form, the former causing death in ten days or less, while the latter may persist for weeks or even months.

In reflex and neurotic vomiting there are no manifest changes in the urine, while the toxemic variety is characterized by a marked decrease in the amount of nitrogen excreted as urea and a characteristic increase in the amount excreted as ammonia. The so-called ammonia coefficient rising from 3 to 5 per cent. to as high as 46 per cent. in one of my cases.

The toxemic type is diagnosticated by the examination of the urine, the reflex by careful bimanual examination of the genitalia, and the neurotic after the exclusion of the other two varieties.

The prognosis is excellent in reflex and neurotic vomiting, provided appropriate treatment is instituted, so that the termination of pregnancy is rarely indicated. In toxemic vomiting, on the other hand, a fatal issue can be averted only by the prompt induction of abortion, and even then the prognosis is dubious.

---

### BOVINE VS. HUMAN TUBERCULOSIS.

At the recent Congress of Tuberculosis, Professor S. Arloing, of Lyons (*Annales de Medicine et de Chirurgie Infantiles*), maintained that:

All tubercular affections result from the Koch bacillus, but this possesses varying degrees of virulence and biological characteristics according to the place where it has lived.

The term "type" ought to be employed only to designate the usual presence of certain characteristics in a bacillus.

All varieties of the bacillus can be agglutinated, can give tuberculin, and therefore can arouse the agglutinating substance in the living body.

The transmissibility of bovine tuberculosis to human tuberculosis and *vice versa* ought not to be discussed; only the frequency of this accident is under discussion.

All tubercular animals should be set apart for the health of man and reciprocally. But it is always the propagation from man to man which is most frequent.

All warm-blooded vertebrates can harbor the Koch bacillus with varying virulence.

The most attenuated varieties of the bacillus in man and in mammals are encountered specially in so-called surgical, gland or skin tuberculosis.

There are, however, cases of surgical or gland tuberculosis which harbor Koch bacilla, as virulent as those of parenchymatous tuberculosis.

By inoculation of guinea-pigs and rabbits we may determine the relative virulence of these germs.

The more alternated varieties may become virulent by passing through the body of a guinea-pig or may preserve their attenuation in spite of many such passages.

---

IN hydrocele the base of the tumor is below, in spermatocele it is usually above. A milky fluid obtained by aspiration usually speaks for spermatocele.—*American Journal of Surgery.*

---

### THE UTERUS AND OVARY OF NEURASTHENIA.

Dickinson observed over one hundred cases of chronic and aggravated type of neurasthenia. The associated lesions in cases of this degree and their frequency would be as follows: (1) In the ovary, chronic oophoritis, chiefly microscopic, was found in nearly all; (2) in the uterus, endometritis, usually cervical, was present in the majority of cases, and was seldom accompanied with thickening; (3) a high degree of sclerosis of the vessels of the uterine walls, and of those of the endometrium was sometimes discovered in cases of long standing, and the venous enlargements were many; (4) about the vulva, certain hypertrophies were noted in two-thirds of the cases; (5) in the bladder, congestion of the trigone was frequent; (6) in the rectum, catarrh, congestion, and atony were persistent in a large number. In this class of cases pelvic symptoms are prominent and lumbar pain constant, and in almost all of the cases pelvic disorder is coincident, not causative. Correction of moderate abnormalities of structure and function by prolonged local treatment or by operation lessens pelvic pain very little and betters the general condition not at all. Treatment should be directed entirely to the general condition: For dysmenorrhœa, bromides with hydrastinin (and helonin); for the menorrhagia, ergot, stypticin; for rectal mucous catarrh, irrigation with astringents and cure of constipation; for bladder irritation, water freely, and urotopin. Besides, training in outdoor life with development of the muscular system, ridding the patient of mental worry or strain, and proper feeding. Operation on pronounced pelvic lesions is warrantable in a few selected cases, such as persistent and exhausting hemorrhages, larger tumors, etc.—*Medical Record, March* 24, 1906.

---

WOVEN catheters may be sterilized by boiling in saturated ammonium sulphate solution. Catheters and bougies may be kept asceptic if they are wrapped in gauze wet with the soap-spirits of the German pharmacopeia.—*American Journal of Surgery.*

---

THE MANNER OF INFECTION IN CEREBROSPINAL MENINGITIS.

A valuable pathological study, based on a series of thirty autopsies has been made by Westenhoffer (*Berl. klin. Woch.,* June 12, 1905). The results of his investigations seem to show that the point of entrance for the infectious germs is the posterior nasopharynx, and particularly the pharyngeal tonsil. The meningitis is at first invariably a basilar process and centers in the region of the hypophysis. It takes place in a lymphogenous manner. The involvement of the cranial cavity is analogous to the inflammation of the mucous membrane of the accessory sinuses connected with the nasopharyngeal space. A meningitis of this type never, or at least very rarely, occurs by the extension of an inflammatory process from the ethmoid cells. The writer claims that both adults and children who happen to be attacked by this disease show distinct evidences of the so-called lymphatic diathesis. The disease is due to the inhalation of the infectious organism and its prophylaxis is essentially hygienic. The meningococcus Weichselbaum-Jäger is found in the majority of cases, although it is by no means decided that it is solely and alone the cause of the disease. The fact that many other occi are found with this coccus, either alone or in a mixture, makes it probable that all of these bacteria play only a secondary role in the etiology of the disease, and that the real etiological factor has not yet been discovered. This seems to be analogous with the streptococcus infection in scarlatina.—*Medical News.*

---

### DOSE OF ANTITOXIN IN DIPHTHERIA.

Fischer *Dietetic and Hygienic Gazette,* January, 1905, states that the proper dose of antitoxin for a mild case of diphtheria should never be less than 2,500 to 5,000 units for a child of any age. For a severe case of diphtheria with marked toxic symptoms, such as a slowness of the pulse and enlarged glands, the dose of antitoxin at the beginning should be between 5,000 and 10,000 units. When a case of diphtheria has been overlooked, and *is seen after the third day of illness,* then 10,000 units should be injected. If the symptoms do not improve during the first twenty-four hours, then 5,000 units should be injected after the first twenty-four hours, and every twenty-four hours until there is a marked improvement in the general condition. If a severe case of *septic diphtheria* is seen after the fourth or fifth day of illness then 10,000 units should be injected. If the symptoms do not improve after twenty-four hours, another injection of 10,000 units should be given. There is no harm done by giving 20,000 units during the first twenty-four hours if a severe case of diphtheria of the nose or a severe case of laryngeal diphtheria is seen. Just as enough calomel or belladonna is necessary to produce the proper systemic effect, likewise antitoxin must be given in proper quantities to neutralize the toxin in the system. If we do not neutralize the toxin in the system by giving the proper antidote, then we must not be surprised to find heart complications (myocarditis), a toxic nephritis or paralysis as a sequela to this disease.

---

### ACUTE GASTRITIS.

In *Merck's Archives* for March, L. R. Barstow states that acute gastritis is not the bugaboo in treatment that the chronic form is. The treatment is confined mainly to a few lines,—rest, both of the organ itself, and of the body; evacuation of the stomach and bowels; control of the temperature and vomiting, and diet. He uses as few drugs as possible. He gives as an emetic and to avoid irritation of the mucous membrane, apomorphin hypodermically, in doses of from one-tenth to one-fifth grain. He finds few patients who are willing to submit to lavage. For evacuation of the bowels, calomel in small doses, one-tenth grain every hour, or calolactose,—being a fine trituration of calomel and bismuth subnitrate, in doses of one grain. This generally controls the vomiting as well. If the latter is very severe, he orders an ice-bag to the epigastrum, small chips of ice by mouth, and finally cerium oxalate, and narcotics such as opium or cocain. In very severe cases, rectal injections of an emulsion containing some form of opium are useful. The temperature is generally easy to control by cool sponging or ice packs, the antipyretics being seldom indicated. As to diet, he prohibits all food for the first forty-eight hours, allowing plenty of cold boiled water, or the carbonated or mineral waters. After this time he commences on a milk diet, four to six ounces every three or four hours, and gradually increases it as the patient's condition improves. If the patient be troubled by sour belching, alkalies, such as sodium bicarbonate, are indicated. With subsidence of the acute symptoms, exhibition of the bitter tonics is generally followed by a prompt recovery.

---

WHEN pruritus ani is caused by a local eczema it is well to remember that the latter may be seborrheal in origin. In such cases other areas of the disease, as on the chest and, especially, the scalp, should be sought for; they will require attention also, in order to effect a cure.—*American Journal of Surgery.*

### THE TREATMENT OF DIFFUSE SEPTIC PERITONITIS.

The author has used Murphy's technic with success in two cases. Murphy reported twenty-nine cases with but one death. The cause of the peritonitis—appendicitis, intestinal perforation, pus tube, etc.—should be eliminated as rapidly as possible without undue handling of the peritoneal surfaces. Drainage of the lowest portion of the pelvis by tube, through a suprapubic opening is practiced; also free drainage through the operative incision. Elimination of all time-consuming procedures is essential. After operation the patient is kept in the semi-recumbent posture of Fowler. To prevent peristaltic movement, all food or liquids by mouth are stopped and opium may be indicated. Large quantities of water are administered per rectum in order to reverse the lymphatic currents of the peritoneum, making this membrane a secreting rather than an absorbing surface and also increasing the secretion of urine. The water is introduced into the rectum by means of a short nozzle, pierced by several openings, and left continually just within the anus. The nozzle is connected with a reservoir placed only a few inches above the level, thus ensuring a constant stream of small velocity and volume. The water trickles in and is absorbed as fast as it is introduced. When not in use the nozzle is disconnected but left *in situ* to reduce the irritation of withdrawal and subsequent reintroduction. As much as nine to twelve pints can be administered in twenty-four to thirty-six hours.—*R. G. Le Conte. Annals of Surgery, February, 1906.*

---

IN dealing with secondary hemorrhage from the rectum (whether bleeding vessels are tied or not), it is better to tampon with gauze wrapped about a piece of stout rubber tubing, than with gauze alone. The tubing allows any further bleeding to show itself externally at once, it allows the escape of flatus, and affords means of introducing an oil injection to facilitate the removal of the tampon later.—*American Journal of Surgery.*

### Scopolamine-Morphine—Chloroform—Anesthesia.

Whitacre reviews the literature on this subject and concludes that:

Scopolamine-morphine narcosis is not devoid of danger.

The use of scopolamine and morphine alone for surgical narcosis is not justifiable, and in my experience is not practicable.

A single dose two hours before operation lessens the discomforts attendant upon the operative procedure to a high degree, and may obtain a definite place in surgical practice.

Four deaths have occurred in a series of 2,400 cases, which have been so definitely related to the use of this method of narcosis that they may be called scopolamine deaths. This, however, in the absence of an autopsy demonstration.

These deaths have been reported as occurring with a type picture of alkaloid poisoning, and heart failure has been given as a direct cause of death. (Landau.)

A fatty degeneration of the liver and kidney has been produced by the use of repeated doses of scopolamine alone and of scopolamine in combination with morphine, in animals.

This method of producing or assisting narcosis can not yet be recommended for use in general practice, in spite of the great advantage it seems to offer. (Kochmann.)—*New York Medical Journal.*

---

### Carcinoma of the Uterus.

Since 1892 the author has operated on over 200 cases for carcinoma of the uterus with a mortality from operation of ten deaths, but in the last seventy-three cases only one patient died. Of 180 of these, which were performed over two years ago, very many have been lost sight of. Of the remainder seventeen are still alive and well at periods varying from twelve years to three years, while 20 per cent. lived and kept free from recurrence for periods varying from two to five years.

When the case is one of uterine cancer the author regards vaginal hysterectomy as the better operation, for the reason that shock to the system is not nearly so severe and patients make a much readier convalescence. Vaginal hysterectomy is also the better operation for those cases in which the disease is limited to the os and vaginal portion of the uterus. When the disease has extended up the cervical canal, infiltrating the cervix and possibly infecting the cellular tissue, then the abdominal route or perhaps the combined abdomino-vaginal operation would be the wiser, if not the only operation which would hold out any hope of success.

With the uterus fixed and the ureters and bladder possibly implicated the author is convinced that they are better left rigidly alone.—*Dr. F. B. Jessett, in Med. Press, February* 21, 1906.

---

### OBSTETRICS IN THE PHILIPPINES.

Bell (*Med. Record*) says that the idea that semi-civilized women escape many of the pangs of childbirth is entirely erroneous in regard to the Philippinos. The Filipino woman lives a short life, owing to her many pregnancies and hard manual labor, insufficient food and most of all the crude, brutal and ignorant practices employed as obstetrical aids. The two chief aids used to assist expulsion of the child are: First, a stout band of cloth passed round the woman's abdomen and pulled tight by four persons, who are seated two on each side of the patient, with their feet against the body. Second, a plank six or eight feet long by a foot wide, which is placed across the woman's abdomen while another person mounted on the plank raises on his toes and descends on his heels forcibly. The birth of the child is followed by the expulsion of the placenta after the above means, and should the process be delayed, forcible traction upon the umbilical cord is made with such energy as to tear away portions of the placenta, and often large sections of this body are left to find their way from the uterine cavity of their own accord. Weeks and even months later the results of such practices are noticed in the septic conditions which should naturally follow retention of the membranes.

### ACUTE AFFECTIONS OF THE PHARANGEAL TONSIL IN EARLY LIFE.

Dupuy, in the *New Orleans Medical and Surgical Journal,* discusses this subject, and reaches the following conclusions:

Head colds in early life are generally characterized by an acute inflammation of the pharyngeal tonsil—the nasal phenomena being purely secondary.

Apart from the many minor ailments caused by acute adenoiditis profound systemic disturbances sometimes follow.

The post-nasal tonsil being a terminal lymph node acts as an avenue of infection for the cervical lymphatic glands; treatment, therefore, of the tonsilar affection offers a very rational protective measure for the prevention of acute lymphadenitis in the region of the neck.

Early and active treatment of recurrent pharyngeal tonsilitis will sometimes prevent a chronic enlargement of this post-nasal lymphatic deposit.

Prophylaxis addressed to the upper air passages will consist essentially in nasal and post-nasal cleanliness, the ideal being attained when the child's nose and naso-pharynx will receive nearly the same attention we bestow on its teeth.

---

THE pain in the lower part of the back that is so frequently complained of after operation, can be best relieved by placing a small pillow in the hollow of the spine.—*American Journal of Surgery.*

---

### PLACENTA PREVIA: A SERIES OF 94 CASES.

The greatest interest in connection with placenta previa is that of treatment. By far the best results which the author has obtained, are those in which the Champetier de Ribes bags were employed. In forty cases the mortality for the mother was zero, and for the child about 60 per cent. When the os is of suitable size, the bag is inserted; it matters little whether the bag is inserted through the placenta or between the latter and the uterus.

In both instances the hemorrhage will be effectively controlled. When the bag is delivered, unless the head or breech is forced down by strong pains, internal version is the best course. The application of the forceps has not been attended with the best results. If the case be one in which the os has already been considerably dilated, internal version or bringing down a leg in pelvic presentations, seems to be the indication. These cases are attended with considerable maternal mortality, because of the great loss of blood that has already taken place. When delivery has taken place, the ordinary methods for combating loss of blood are in order. The author thinks highly of rectal saline infusions. —*Richard Warren in Lancet, February 3, 1906.*

---

IN crying infants it is extremely difficult to determine the presence, and location, of tender areas. This may be readily accomplished by the administration of chloroform to the extent of *primary* narcosis. The physical examination then becomes very easy, and when the tender spot is handled it will be announced at once by lively reflexes.—*American Journal of Surgery.*

---

### TO PREVENT YELLOW FEVER.

Since the recent epidemic of yellow fever in New Orleans much work has been done to lessen the possibilities of another outbreak of a like nature in the towns on the Southern coast. Acting Assistant Surgeon Sheely, of the United States Public Health and Marine Hospital Service, in a recent issue of Public Health Reports, urges the following: (1) Removal of stagnant water and all receptacles capable of affording breeding-grounds for the stegomyia; (2) screening and oiling of all cisterns before March 1; (3) the efficient screening of all houses with screens not less than 16 meshes to the inch, by the same date; (4) provision of at least one mosquito-proof screen to be used on the first sign of any febrile disease; (5) a general fumigation of every house on one of the coldest days in February, so as to kill the stegomyia in winter quarters. The rigid following of these rules would make an outbreak of yellow fever an impossibility.—*Medical Age, April, 1906.*

---

### THE VALUE OF DRUGS IN THERAPEUTICS.

Shattuck states the leading therapeutical principles as follows: Do no harm. This principle seems to be well met by the homœopathist, who uses the infinitesimal dose. He does no harm, save in so far as he may miss doing good. Try to see as clearly as possible just why you give a drug, your purpose in giving it, whether as a specific, curative, palliative, or as a placebo. As far as you can, give a drug uncombined. This is a general rule subject to many exceptions. In using an efficient drug be as sure as you can of a good preparation, and then give it until something happens, either the desired effect, or evidence appears that the limit of toleration has been reached, what is called the physiological, but what the author calls the toxic effect. Disregard of this law is responsible for many therapeutical failures. Our knowledge of drugs as therapeutical agents rests mainly on an empirical basis, and in drawing conclusions we must be keenly alive to the manifold sources of error.—*Boston Medical and Surgical Journal, March 29, 1906.*

---

### TEMPERATURE DURING MENSTRUATION.

E. Frank *(Berliner klin. Woch.)* calls attention to his previous announcement, that fever during menstruation is a symptom of some morbid process in the body. It is especially indicative of tuberculosis, and if the patient is anemic, with a tendency to sweat, antitubercular treatment should be inaugurated. The author states that the normal temperature never exceeds 99.5° F., and even a fraction of a degree above this is to be regarded as fever. Sabourin and Kraus have also reported this condition, and state that if inflammatory conditions of the adnexa can be excluded it is indicative of tuberculosis.—*Medical Age, February 10, 1905.*

### SURGICAL TREATMENT OF PARALYSIS.

The types of cases discussed by Tubby are those arising from anterior poliomyelitis, spastic paralysis, ischemic paralysis and some traumatic lesions of the nerves. The modern methods of treatment discussed are tendon and muscle transplantation, arthrodesis and nerve anastomosis. Among the cases reported by Tubby are the following: Grafting the extensor proprius pollicis into the tibialis anticus, and part of the extensor longus digitorum into the internal cuneiform bone for paralytic equino-valgus associated with paralysis of the tibialis anticus; partial paralysis of the extensor cruris; insertion of the ilieo-tibial band into the patella, which was followed by complete recovery of the power of extension of the leg; grafting the distal facial trunk for traumatic facial paralysis into the hypo-glossal.—*British Medical Journal, March 3, 1906.*

---

IF, after a period of post-operative catheterization, the patient finds herself unable to pass urine spontaneously, apply hot towels to the vulva.—*American Journal of Surgery.*

---

### THE EFFECT OF SMALL QUANTITIES OF ALCOHOL UPON THE HUMAN BRAIN.

In a recently published lecture by Victor Horsley, the statement is made that "one prominent defect in the study of the alcohol question has always been the want of scientific proof that small quantities of alcohol, such as are used every day in ordinary diet, have any appreciable adverse effect upon our organism. That defect has now been supplied. We have it definitely established by the precise and more delicate investigations of the last ten or fifteen years that it is indeed the case. From a scientific standpoint, therefore, the contention, which we have so often had put before us by our friends, that small doses of alcohol, such as people take at meals, have practically no deleterious effect, can not be maintained.—*Review in Glasgow Medical Journal, March, 1906.*

GROWTH and achievement in the medical world are proportionate in a large measure to the amount of intelligent reading done systematically day by day. Commenting upon the work of one of the most progressive young practitioners in the South, Dr. William J. Mayo recently remarked: "Yes, every time I see him, he is bigger than when I saw him last."

The factors in such development are many. Opportunity is much; inate faculties are more, for they often create opportunity, but the basic principle which must underlie all permanent success in practice involves knowledge; knowledge not only of present facts and theories, but of every advance—of every intimated discovery or revelation; discriminating knowledge which quickly separates wheat from chaff—and garners it to the storehouse.

This involves diligent and daily perusal of medical books—new and old; of the best medical journals—and only the best in these—for every journal contains more or less chaff; and, lastly, the setting apart each day or night an hour when least likely to be disturbed—for a mental review of the day's work—the searching of literature for light on every case—that one's service to humanity may be the best, and that to the rich experience of each day's work may be added a certain permanency of increased knowledge—classified and detailed in an hour of quiet thought—and corroborated and brought up to date by touching the high places in the latest and sanest literature. Many medical men religiously purchase new medical works—for the mental effect upon their patients— and tolerate medical journals for their decorative touch to the table in the waiting-room. But the man who is bigger every time you meet him than when you saw him last—jealously sets aside each day a time for thought and study and mental acquirement.—*Editorial, Southern Medicine and Surgery.*

---

VAGINAL cleanliness has been studied by Ahlfeld (*Zeitschr. f. Geb. u. Gyn.*, Vol. 54, No. 1), with regard to the question of the effect which the preliminary cleansing of the vagina has on the morbidity of the puerperium. No disinfecting effect can be obtained with a one per cent. solution of cresol; at least three per cent. is essential for efficient irrigation. The average of his "irrigated" cases in which no temperature occurred was about 68 per cent.; while during two years in which no irrigations were employed the number sank to 53 per cent. In operative cases the non-fibrile average was even greater than above stated; here the cleansing was more complete and thorough. When streptococci were demonstrated in the vagina before douching, a marked decrease in the number of growing organisms was noticed within a few hours after the irrigation had been given. Ahlfeld declares that as irrigation is attended with such good results it should be much more frequently employed. Here we thoroughly agree with him; but it is well, we think, to mention the scientific fact that of all normal bodily secretions those of the vagina are perhaps most inimical to micro-organisms.

---

### COLORLESS IODIN.

Here is a very easy way to make colorless iodin:

℞ Iodin.............................................. ℥ vij.

Aqua Ammonia.................................... ʒ iss.

Carbolic Acid...................................gtt. x to xij.

Shake well and wait just a moment and all color will be gone. If the aqua ammonia is old, or the carbolic acid old, the change probably will not be instantaneous. The change may come slowly, but not absolutely transparent with old aqua ammonia or old carbolic acid. The therapeutic value of the iodin has not been harmed at all for local application. In fact it can be used with better results to reduce a swelling than the iodin before being changed.—*C. E. Hoover in the Medical World.*

---

IN performing posterior gastro-enterostomy see that the opening in the transverse mesocolon is not so narrow that it may constrict the anastomosed segment of small intestine nor yet so large that it may permit of a possible hernia into the lesser sac. By inserting a number of sutures between the mesocolon and the stomach wall about the asastomosis these possibilities may, in large part, be obviated.—*American Journal of Surgery.*

### EXPERIMENTS ON THE TRANSMISSION OF SYPHILIS TO APES.

Neisser and his colleagues experimented in Batavia in order to exclude the harmful influence of a cold climate. He made use of orang-utans, gibbons, and macacos. The progress of the transmitted infection was modified by the diseases which were attributable to captivity, but, notwithstanding, there were some remarkable results. The incubation period varied from fifteen to sixty-five days, depending upon the quantity and freshness of the infecting material. The latter was taken from human beings in various stages of the disease. Human blood and serum did not transmit the disease. Inoculation from animal to animal was effective with material taken from chancres, from the spleen, spinal cord, glands, and testicles. Inoculation was possible by inunction upon scarified tissues. Secondary syphilitic phenomena occurred only with gibbons. The introduction of mercury and iodine did not prevent the development of the disease after the poison had been introduced. Neisser thinks it very probable that the cause of syphilis resides in the spirocheta, but he failed to find it in a series of fresh cases in both men and apes.—*Fortschrift der Medizin, February, 1906.*

---

IN strapping the chest for fractured rib, two points should be particularly noted: 1. The straps should pass well beyond the median line. 2. They should be applied in full expiration. One or two straps passed over the shoulder help much to secure immobilization.—*American Journal of Surgery.*

---

CHRONIC rheumatism is defined by Teissier (*Le Bull. Medic.*) as an infectious disease, the pathogenic agents being multiple and varied in different cases. Thus the disease shows itself in diverse forms. The osteoarticular type includes primary or secondary chronic rheumatism, certain forms of spondylosis (which are, however, rare), characterized by deforming articular lesions, and certain cases of Heberden's nodes. The serous type includes: cases of chronic synovitis and tendovaginitis; some latent pleurisies; and (in general) such skin phenomena as purpura, erythema multiforma, and erythema nodosum. The fibrous type is seen in the hypertrophy of periarticular organs; in some cases of Dupuytren's disease, of spondylosis with alterations in the vertebral ligaments, and in scleroderma. The muscular type is seen in chronic rheumatismal myositis and some cases of spondylosis of muscular origin. The existence of tuberculosis rheumatism is merely hypothetical.

---

FREQUENTLY referred to the surgeon because of the constant pain and marked tenderness, is to be noted a group of cases of what might be termed *occupation wrist pain.* They differ from the ordinary case of "writer's cramp," "piano-player's cramp," etc., in that, while these latter frequently have pain in, or about, the wrist, the cases here referred to have no spasm, the pain is constant, and it is not of a neuralgic character. Sometimes it radiates along the thumb (as in mail-openers); sometimes it is localized to the inner border of the lower end of the ulna, which is very sensitive to pressure (as in shirt-ironers). The fingers are free. There may be pain in the forearm muscles (flexors).—*American Journal of Surgery.*

---

POST-OPERATIVE hemorrhage from the base of the bladder that proves inaccessible to ligatures, and uncontrollable by packings, may be checked by the following method: Through several thicknesses of gauze, cut in squares, pass a double strand of heavy silk or of twine fastened on a stout needle. With the patient in Trendelenburg's position and the bladder widely opened, thrust the needle from within directly through the perineum, and bring the gauze firmly against the bleeding surface by pulling upon the threads, which are then to be fastened to an outside dressing.—*American Journal of Surgery.*

---

WHEN performing external urethrotomy without a guide it is often possible to trace the continuation of the urethra proximal to the opening, by means of a filiform bougie, even when all devices failed to secure the introduction of a filiform before the operation. If a filiform can not be thus passed through the urethral wound, suprapubic pressure on the bladder may demonstrate the location of the urethral orifice by the escape of a drop of urine or by bulging of the membranous urethra.—*American Journal of Surgery.*

---

EDUCATION IN THE ORIENT.

Among its miscellaneous minor articles, *The Open Court* for October contains three which deal with the problem of education as it is presented in different parts of the Orient. One describes the new elementary text-books which are to introduce modern ethical principles into the minds of the youthful Japanese.

---

DURING the conduct of a narcosis, more important than the activity of the conjunctival reflex or the actual size of the pupil in determining the depth of the anesthesia, are the *changes* in the reactibility of the lid and the alterations in the size of the pupil. They are reliable indices to fluctuations in the depth of the narcosis. Sometimes a patient is quite relaxed and anesthetic although a fair conjunctival reflex is present; and, again, it may occasionally happen that a patient reacts even when that reflex is abolished.—*American Journal of Surgery.*

---

ALTHOUGH the anal reflex requires profound anesthesia to abolish, chloroform or ether is not always needed in order to divulse the sphincter ani. This may be accomplished painlessly, and usually with entire satisfaction, under ethyl chlorid or nitrous oxid narcosis if, especially, an opium suppository is introduced a half hour beforehand, and a pledget of cotton wet in cocain solution is applied just before the operation.—*American Journal of Surgery.*

---

Do not advise extirpation of large glands in any particular region without making sure that they are not the early manifestations of leukemia or Hodgkin's disease.—*American Journal of Surgery.*

---

IN all operations in the left subclavian triangle of the neck, the location there of the thoracic duct must not be forgotten.—*American Journal of Surgery.*

